# Human Papillomaviruses-Related Cancers: An Update on the Presence and Prevention Strategies in the Middle East and North African Regions

**DOI:** 10.3390/pathogens11111380

**Published:** 2022-11-19

**Authors:** Queenie Fernandes, Soumaya Allouch, Ishita Gupta, Ibrahim Elmakaty, Khaled E. Elzawawi, Ahmed Amarah, Hamda Al-Thawadi, Halema Al-Farsi, Semir Vranic, Ala-Eddin Al Moustafa

**Affiliations:** 1 College of Medicine, QU Health, Qatar University, Doha P.O. Box 2713, Qatar; 2 Translational Cancer Research Facility, National Center for Cancer Care and Research, Hamad Medical Corporation, Doha P.O. Box 3050, Qatar; 3 Research Department, Sidra Medicine, Doha P.O. Box 26999, Qatar; 4 Biomedical Research Center, Qatar University, Doha P.O. Box 2713, Qatar

**Keywords:** human papillomaviruses, cervical cancer, colorectal cancer, head and neck cancer, breast cancer, the Middle East and North African region

## Abstract

The human papillomavirus (HPV) is a non-enveloped double-stranded DNA virus capable of infecting skin and mucosa epithelial cells. Commonly, HPV infection is associated with sexually transmitted diseases and is considered the leading cause of cervical cancer and other carcinomas of the anogenital tract. However, several studies reported their involvement in cancers of non-sexual regions, including colorectal, head and neck, and breast cancers. There are several studies from the Middle East and North Africa (MENA) regions on the potential association between high-risk HPVs and cancer; nevertheless, there are limited studies that address the significance of HPV vaccination as a potential guard against these cancers. In the current review, we present a comprehensive description of the current HPV-associated cancers prevalence rates in the MENA region, demonstrating their steady increase with time, especially in African regions. Moreover, we discuss the potential impact of vaccination against HPV infections and its outcome on human health in this region.

## 1. Introduction

Cancer is one of the leading causes of mortality, with approximately 20 million new cancer cases worldwide and 10 million cancer deaths [1]. Along with environmental and genetic factors, recent studies attribute 20% of human cancers to viralor bacterial infections [2]; with several reports revealing the presence of viruses in both solid and non-solid tumors [3]. Some of the most commonly identified viruses include Epstein–Barr virus (EBV), human papillomaviruses (HPVs), human herpes virus 8 (HHV8 or Kaposi’s sarcoma-associated herpesvirus), as well as hepatitis viruses B and C (HBV and HCV) [3]. Advancement in molecular biology showed viral oncogenes to alter cell signaling and growth control pathways, thus triggering the onset and development of human diseases, including cancer [4].

Human papillomaviruses (HPVs) are small, double-stranded DNA viruses with an icosahedral capsid belonging to the *Papillomaviridae* family and are the etiological agent of dermatological and sexually transmitted diseases [5]. Commonly, HPV is the most common cause of sexually transmitted infections worldwide, with once in a lifetime infection risk amongst both men and women of about 50% [6]. HPVs have the ability to infect the cutaneous and mucosal epithelial tissues of the skin, upper respiratory, and anogenital tracts [5]. Although HPVs are well-known inducers of common and anogenital warts [7], persistent infection with high-risk HPVs is associated with several human carcinomas, especially cervical cancer [5,8].

More than 200 HPV types are identified. Based on the homologous nucleotide sequence of their L1 protein, a phylogenetic tree was generated that classifies HPV types into five genera, α, β, γ, μ, and ν [9,10]. The α (mucosal) genus is the most studied and consists of around 30 HPV types associated with the infection of the mucosal epithelial tissues of oral and anogenital tracts and benign cutaneous HPV types involved in the development of skin warts. Depending on their oncogenic ability, mucosal HPV types are classified into low-risk (LR) and high-risk (HR) HPVs. Infections induced by low-risk HPVs (HPV types -6 and -11) include recurrent respiratory papillomatosis, benign gynecological papillomas, and skin warts [11,12,13]. On the other hand, high-risk HPVs (types -16, -18, -31, -33, -35, -39, -45, -51, -52, -55, -56, -58, -59, -68, -73, -82, and -83) have oncogenic potential and are associated with the development of human cancers [9]; HPV 16 and HPV18 are frequently present in cervical cancer where more than 96% of these cancers are positive for these viruses [14,15]. Subsequently, high-risk HPVs are shown to be involved in a subset of other genital cancers in addition to head and neck, colorectal, and breast cancers [16,17,18,19,20]. Several studies investigated the presence of HPVs in cancer and found the presence of high-risk HPVs correlated with vascular invasion, lymph node metastasis, and tumor grade and size [21,22,23,24]. On the other hand, several in vitro and in vivo studies reported oncogenic properties of the β HPV genus [25,26,27,28]; nevertheless, the γ, μ, and ν genus are found to be involved with skin tropism with no documented role in oncogenesis [5].

The HPV genome is approximately 8 kb in size and consists of 8 or 9 open reading frames (ORFs) located on the same DNA strand [29]. The HPV genome is divided into three different regions, including an early region consisting of the early genes (E1, E2, E4, E5, E6, and E7), a late region containing the late genes (L1 and L2), and a non-coding region (long control region (LCR)) located between ORFs L1 and E6; the LCR majorly consists of regulatory components in charge of viral DNA replication and transcription [29]. The E1 and E2 genes encode viral DNA replication proteins and are initially expressed upon viral entry into the host cell [30]. Along with E1, E2, and E4 replication proteins, the E5 protein promotes viral DNA replication in low copy numbers [31]. However, during epithelial cell differentiation, the p670 promoter enhances E1, E2, E4, and E5 proteins and triggers L1 and L2 expressions, resulting in virion production and increased viral DNA amplification leading to a hyperproliferative state [31,32]. Moreover, the oncoprotein E5 can promote cell transformation and induce carcinogenesis via pro-apoptotic proteins and EGF-R1 signaling pathways (MAP kinase and PI3K-Akt) [33,34,35]; while early oncoproteins, E6/E7, regulate cell cycle and inhibit host-triggered apoptosis, either by downregulating the expression of TNF-R1 or via p53 inactivation [36], thereby presenting alternating mechanisms of HPV-induced oncogenesis [37,38]. More specifically, E6 binds to pro-apoptotic proteins, such as Bax and Bak [39,40,41]. It also binds and blocks the activity of tumor suppressor molecules, such as pRB and p53 [38,42,43]. Additionally, E6 can enhance telomerase activation, resulting in altering pathways regulating cellular proliferation, differentiation, immune recognition, and survival signaling [38]. Moreover, independent of E7, E6 binds to PDZ via its C-terminal, leading to normal cell-to-cell adhesion disruption, thus, inducing suprabasal cell proliferation, an important step in the development of metastatic tumors [44,45]. From its side, E7 increases genomic instability by binding to pRb and displaying E2F, leading to the accumulation of chromosomal abnormalities, further creating a suitable environment for the neoplastic transformation of cells [46]. In our laboratory, we reported that E6/E7 cooperate with ErbB-2/HER2 receptors to trigger cellular transformation through D-type cyclins (D1, D2, and D3) [47,48,49] via β-catenin tyrosine phosphorylation by pp60 (c-Src) kinase activation [50,51,52]. Additionally, E6/E7 oncoproteins enhance the expression of fascin, Id-1, and P-cadherin, leading to increased cell proliferation, invasion, and metastasis [47,49,53,54,55,56]. Alteration of EGF-R1 and Id-1 expression triggered by E5 along with E6/E7 oncoproteins indicate a plausible cooperation of HPV oncoproteins in the onset and development of human cancers by stimulating epithelial-mesenchymal transition (EMT) [57]. The HPV cycle is completely intraepithelial, non-lytic, and blocks pro-inflammatory signal activation, thus recruiting antigen-presenting cells and releasing cytokines; these events promote cell growth and proliferation [5]. Nevertheless, most high-risk HPV types evade host immune recognition and induce persistent infection, promoting neoplastic transformation and cancer progression [5].

Numerous investigations worldwide reported a varying prevalence of HPV infection subtypes in human cancers, indicating an association between geographical location and HPV prevalence [15,18,58,59,60]. The Middle East and North Africa (MENA) region consists of 19 countries, including Algeria, Bahrain, Egypt, Iran, Iraq, Israel, Jordan, Kuwait, Lebanon, Libya, Morocco, Oman, Qatar, Saudi Arabia, Syria, Tunisia, United Arab Emirates (UAE), and Yemen. Though the MENA region is highly diverse politically, culturally, and economically, most of the population in the MENA region shares conservative cultural values [61]. As compared to the West, the MENA region has a lower HPV prevalence rate [62]. However, most MENA countries do not screen for HPV infections due to stigmatization based on religious and traditional values, plausibly leading to small sample sizes and low incidence rates of HPV infection, thus miscalculating the actual number of cases [62].

Moreover, since HPV infection is majorly sexually transmitted, the introduction of vaccination programs within countries of the MENA region is nascent [61,63]. Today, in addition to Turkey (which includes the organized screening program) [64], Libya and UAE are the only two countries with HPV vaccine included in their health programs [62]. On the other hand, countries such as Qatar, Algeria, and Morocco included HPV vaccination programs in their health settings, but they are not readily available to the population [62].

As compared to developed countries, reports on HPV infection and human cancers are limited in developing countries, including the Middle East and North Africa (MENA) region. Herein, we will review the updated literature on the presence and distribution of HPVs in human cancers with a special focus on cervical, head and neck, colorectal, and breast cancers in the MENA region. The countries of the MENA region will be classified into the GCC (Bahrain, Kuwait, Oman, Qatar, Saudi Arabia, and UAE) and Yemen region, Levant countries (Iraq, Jordan, Lebanon, Palestine, and Syria), Maghreb countries (Algeria, Libya, Morocco and Tunisia) and Other countries of the MENA region (Egypt, Iran and Israel). Turkey, a country of unique Ottoman descent, is often listed among Middle-Eastern countries since it occupies regional territories in both Asian and European continents, sharing boundaries with other Middle-Eastern-Mediterranean countries, such as Syria and Iran; thus it will also be included in the list of the countries discussed.

## 2. High-Risk HPVs in Cervical Cancer

Cervical cancer is the fourth most commonly diagnosed cancer, as well as the fourth leading cause of cancer death in women [1]. Infection with high-risk HPV is the most common cause of the onset and development of cervical cancer and accounts for more than 99% of all cervical cancer cases [65]. Although a non-sexual mode is reported, sexual transmission remains the most common mode of infection [66]. Following high-risk HPV infection, the virus is deposited on the basal layer of the cervical epithelium infecting its mucosa [66]. Viral replication occurs, and the HPV DNA is integrated into the cellular genome, leading to the onset of cervical cancer [66]. On average, persistent HPV infection progresses into high-grade cervical intraepithelial neoplasia and invasive cancer within 15–30 years [67].

In approximately 50% of the cases, high-risk HPV-infected women do not develop clinically detectable serum antibody levels and thus pose a risk of re-infection with the same HPV subtype [68]. Today, widespread HPV infection and its association with cervical cancer made HPV vaccination an essential part of cervical cancer prevention strategies. HPV vaccines produce continuous levels of serum-neutralizing antibodies, which are efficient in alleviating the disease [68]. Formal screening and vaccination programs in high-income countries helped reduce cervical cancer incidence and mortality by 50% [67]. However, in the middle- and low-income countries, the prevalence of cervical cancer is still high (~90%) due to the lack of HPV screening and vaccination programs [67]. To date, three HPV vaccines (Cervarix, Gardasil, and Gardasil-9) protecting against either two, four, or nine types of HPV, respectively, protect against HPVs -16 and -18, which are the most frequent high-risk HPV subtypes, in addition to other HPV types [69,70], and are effective in preventing cervical cancer development by 70% [71]. A nationwide study from Sweden by Lei et al. (2020) [23], including over 1.6 million females aged 10 to 30 years, found that HPV vaccination correlates with a reduction in the incidence of invasive cervical cancer. Despite its effectiveness, HPV vaccination coverage worldwide is still limited to 15% [72].

Nevertheless, though the association between HPV infection and progression of cervical cancer is well known, only a few studies from the MENA region reported data on HPV-associated cervical cancer (Table 1). A meta-analysis by Obeid et al., 2020 [62] reported a pooled HPV prevalence rate of 81% in cervical cancer patients, 54% in women with abnormal cervical cytology results and 16% among the general population in the MENA region. Moreover, the study reported the highest HPV positivity in cervical cancer in Maghreb countries (88%), while the lowest prevalence was found in Iran (73%) [62]. On the other hand, abnormal cervical cytology subgroup had a pooled HPV prevalence of 54%, with the highest reported prevalence in Northeast Africa (94%) and the lowest in the Levant region (31%) [62]. Furthermore, middle- and low-income countries had the highest HPV prevalence rates [62]. Similar to other regions, in the MENA region, the most commonly detected HPV subtype in cervical cancer samples is HPV16, followed by HPV18 [62]. Another study also evaluated the pool prevalence of HPV and its high-risk genotypes in Middle Eastern countries and reported a prevalence of 12.3% and 5.2%, respectively [73]. Below, we provide the prevalence and distribution of HR-HPVs and their association with cervical cancer in MENA countries (Table 1).

### 2.1. In GCC Countries and Yemen

In the GCC region, HPV positivity was reported in ~10% of cervical cancer patients; high-risk HPVs were present in only ~4% of the samples [73]. A recent study by Ali et al. (2019) [74] analyzed the presence of cervical high-risk HPV infection among women in some GCC countries, including Bahrain, Saudi Arabia, Qatar, and UAE. The study reported 21% of cervical high-risk HPVs in women, of which Arab women had a significantly lower prevalence of high-risk HPV infections compared with non-Arab women (16.4% vs. 31.6%); however, no significant difference in the distribution of high-risk HPV infection types was present between the two groups [74]. Demographically, the overall high-risk HPV prevalence was highest in women from Qatar (31%), followed by women from Bahrain (20%), Saudi Arabia (17%), and UAE (15%) [74]. Surprisingly, younger women (<50 years) were positive for high-risk HPVs, indicating that younger women are at a higher risk of HPV infection [74]. In addition, while infection with HPV16 and HPV18 was low (17% and 3%, respectively), infection with other high-risk HPVs was highly predominant, accounting for 64% of the cases [74]. Based on the cytological diagnosis, the overall high-risk HPV positivity rate among women with normal and abnormal cytology was 15% and 51%, respectively, indicating that the presence of high-risk HPVs is associated with abnormal cytology [74]. The study also evaluated the presence of high-risk HPV positivity in women with atypical glandular cells not otherwise specified (AGC-NOS), atypical squamous cells that cannot exclude high-grade squamous intraepithelial lesion (ASC-H), atypical squamous cells of undetermined significance (ASCUS), high-grade squamous intraepithelial lesion (HGSIL), low-grade squamous intraepithelial lesion (LGSIL), and negatively for intraepithelial lesion or malignancy (NILM) [74]. The results indicate an increase in high-risk HPV positivity rates with the severity of cytological lesions, from 41% in women with ASCUS to 60% in those with HSIL as compared to (15%) in NILM [74].

In Bahrain, a 10–12% prevalence of HPV-DNA was reported in cervical cancers [75,76,77]. While one study detected HPVs -52, -16, -31, and -51 and HPVs -6, -70, and -74 to comprise the high-risk and low-risk subtypes, respectively [75], another study reported HPVs -16, -18, -45, -62, and -53 [76]. Moreover, higher high-risk HPV type prevalence was reported in women below 25 years of age [75].

On the other hand, a higher prevalence of HPV was reported in Kuwait; as compared to normal cervix cytology, women with abnormal cytology had higher HPV positivity (2% v/s 51%) with a higher incidence rate in younger women (<50 years) [78,79]. Al-Awadhi et al. (2013) [79] reported the presence of HPV in women with invasive cervical carcinoma (86%), high-grade squamous intraepithelial lesion (67%) and low-grade squamous intraepithelial lesion (89%), respectively. Moreover, genotyping revealed the predominant presence of HPV16 (24%) in women with abnormal cytology, followed by HPVs -11, -66, -33, -53, -81, -56, and -18 [79].

Notably, to date, only one study was performed in Oman regarding the presence of HPV in women with normal and abnormal cytology; the study reported a prevalence of 17% and 37.5% of HPV, respectively [80]. The study further reported the presence of 22 different HPV genotypes (15 high-risk and seven low-risk), with HPV82 (high-risk, ~11%) and HPV54 (low-risk, 12%) being the most prevalent among women [80].

Compared to the Omani population, in Qatar, varying prevalence of HPV was reported. While earlier investigations revealed a lower HPV prevalence rate (6–8%) [81,82], recent studies reported a high HPV prevalence rate (64%) [83]. In this regard, Elmi and colleagues [82] further evaluated the levels of IgG and IgM and found an overall HPV-IgG seroprevalence rate of 4.5%. Meanwhile, Al-Thani et al. (2010) [83] reported HPV prevalence based on cytological diagnosis; 54%, 86%, and 50% of women with ASCUS, LGSIL, and HGSIL were HPV DNA-positive, respectively, and 67% with squamous cell carcinoma were HPV DNA-positive. Moreover, a high prevalence of HPVs -16, -18, -52, -56, and -59 were reported as the common high-risk HPV genotypes [81,82,83], and HPV81 as the most predominant low-risk genotype [81].

Amongst the general population of Saudi Arabia, a 9.8% prevalence of HPV was present in the general population [84]; studies combining Pap tests and HPV screening in women revealed HPV positivity in 3–32% of the cases [85,86,87,88]. Nevertheless, in cervical cancer in Saudi Arabia, studies reported HPV positivity ranging from 43 to 95% with -16, -18, -31, -33, -45, and -73 being prevalent high-risk HPV subtypes [89,90,91].

On the contrary, UAE reported the highest prevalence of high-risk HPVs (88%) in samples from women diagnosed with cervical squamous intraepithelial neoplasia and cervical carcinomas, with HPV16 as the most commonly detected HPV subtype [92]. Similarly, another study involving women diagnosed with ASCUS reported the presence of high-risk HPVs in 18% of the cases, especially in pre-menopausal women with HPV16 as the predominant subtype [93]. HPV18 was reported as a less frequent high-risk HPV subtype [93,94].

Similar to HPV screening in GCC countries, where the prevalence of HPV was established, in Yemen, studies report the prevalence of HPV subtypes in cervical cancer. Bensumaidea and colleagues [95] examined cervical cancer tissue samples and reported 74% and 28% of the cases to be positive for HPV16 and HPV18, respectively. The same group further examined cervical cancer tissue samples in comparison with benign cervical lesion tissue (control) samples reporting the prevalence of HPVs -52, -56, -58, -59, and -66; in addition to HPVs -51 (0.6%), -58 (4%), and -59 (3.3%), the authors did not find HPVs -56 and -66 present among the cervical cancer cases, while only HPV 58 was identified in the controls (2%) [96]. On the other hand, a study conducted on cervical cancer samples from Yemeni women reported 24% of HPV positivity with prevalent subtypes, including HPV31 (7%), HPV33 (4%), HPV35 (4%), HPV39 (3%), and HPV45 (7%) [97].

### 2.2. In Levant Countries

Similar to studies in GCC, studies in the Levant countries are also limited. In Iraq, Goral et al., 2019 [98] and AlMufty et al., 2016 [99] explored cervical HPV genotype distribution among different Pap readings. Intriguingly, Goral et al. reported 40.6% HPV-positive cases, out of which 46.2% were normal, and 53.8% had abnormal Pap smears. Additionally, both high-risk (HR-HPV) and low-risk (LR-HPV) genotypes were identified in the sample with a higher frequency for HR-HPV [98]. Concurrently, AlMufty et al. tested 104 samples obtained from women with vaginal discharges and lower abdominal pain, 12.5% were positive for high-risk HPV genotypes, out of which 30.7% were HPV16, and 53.8% showed mixed genotyping [99]. Jubrael et al., 2012, studied the levels of IL-10 and TNFα cytokines in women with HPV DNA^+^ and DNA^−^ cervical lesions [100]. The authors reported a significantly higher level of IL-10 in cervical secretions of HPV DNA^+^ compared to DNA^−^ patients (88.73 vs. 24.00 pg/mL) and in samples obtained from healthy individuals (control) (88.73 vs. 8.27 pg/mL). On the other hand, no difference was noted in the levels of TNFα in cervical secretions of HPV DNA^+^ vs. DNA^−^ cases and controls (12.18 vs. 9.90 vs. 7.90 pg/mL, respectively) [100]. The study suggests that the notable increase in IL-10 compared to TNFα could be due to a down-modulation of tumor-specific immune responses to HPV-infected lesions [100].

Two Jordanian studies reported the presence of HPV infection in cervical cancer; they show an increase in the prevalence of HPV infection in Jordanian women with cervical cancer [101,102]. The first study reported an overall high-risk HPV prevalence in cervical cancer (87%), low-grade squamous intraepithelial lesions (72%), and high-grade squamous intraepithelial lesions (79%); genotyping identified HPV16 as the most predominant subtype (54%) in all cervical lesions [101]. A recent analysis by Abu-Lubad et al. (2020) [102], reported an increase in HPV infection in cervical cancer patients against controls (92% v/s 61%); this study also reported significant dominance of the HPV16 subtype. Contrary to findings from GCC countries, in Jordan, women older than 50 years of age displayed the highest HPV infection rate [102]. Compared to Jordan, in the Lebanese population, only one study was carried out to determine the presence of HPV in Lebanese women with cytological abnormalities; the study reported the presence of HPV in 5% of the women with HPV16 as the dominant subtype [103].

Similarly, in Syria, a high rate of high-risk HPVs was reported in cervical samples (95%) with HPV33 as the most prevalent high-risk HPV, followed by HPVs -16, -18, -45, -52, -58, -35, -51, and -31 [104]. Moreover, the study revealed that E6 expression of high-risk HPVs correlates with overexpression of Id-1, indicating the progression of HPV-positive cervical cancer via Id-1 regulation [104].

### 2.3. In Maghreb Countries

In Algeria, Mougin et al., 2016 reported a 100% prevalence of HPV in cervical cancer samples [105]. On the other hand, Mouhammedi et al., in 2017, examined cervical samples from 96 women that were either HIV-1-infected or presenting with a gynecological disease [106]. The authors found that 60% of tested samples were HPV+, out of which, HR-HPV infection accounted for more than 80%. Remarkably, the study also noted a significantly higher frequency of HPV infection among the HIV-1-infected group [106].

In Libya, only one study was published in 2014 exploring the prevalence of HPV in North-Eastern Libyan patients with cervical cancer [107]. The study reports a strong connection between HPV and cervical cancer, with the majority (82.5%) of tested samples positive for HPV-16 and 12.7% positive for HPV-18 genotype [107].

In Morocco, various studies were published between 2010 and 2017, investigating the prevalence and genotype distribution of HPV in cervical cancer [108,109,110,111]. HPV infection in patients with cervical cancer was reported to be 92.5% versus only 13.9% in healthy cases, with HPV16 as the most common genotype, followed by HPV18 [111]. Concurrently, Elgnaoui et al., 2016, [110] reported that 74% of tested biopsies were positive for HPV, of which 67.7% were high-risk HPVs, and 24% were low-risk HPVs. In Fez and neighboring areas, the reported prevalence of HPV was only 43.1%, with the most prevalent genotype being HPV 53 [110].

In Tunisia, a recent study investigated the distribution of HPV in precancerous and cancerous cervical neoplasia and reported an HPV prevalence of 83% in cervical lesions [112]. More specifically, HPV was detected in 65% of CIN I, 82% of CIN II/CIN III, and 85% of cervical cancer cases [112]. Genotyping revealed the presence of HPVs -16 and -18 in all tested lesions, and a significant association between HR-HPV and cervical intraepithelial neoplasia was determined [112].

### 2.4. In Other Countries of the MENA Region and Turkey

In Egypt, Yousef et al., 2016 [113], detected 26 HPV types with a prevalence of 40.8% in the 152 women tested. In cytologically normal females, HPV prevalence was 17.7%, 56.5% in patients with a low-grade squamous intraepithelial lesion (LSIL), 3.2% in the high-grade squamous intraepithelial lesion (HSIL), and 22.6% in invasive squamous cell carcinoma (SCC) groups [113]. Similarly, HPV positivity of 22% was reported in cervical cancer patients with a prevalence of 6.5% of high-risk HPV subtypes [73]. Recently, Elazab, M et al., 2021, reported that out of 1000 women included in the study, only 14.3% were tested for HPV and were found positive, while 85.7% of women were not tested. In addition, 67.8% of HPV-positive women were found to have different degrees of CIN [114].

In Iran, Khodakarami and colleagues [115] were the first to report HPV positivity in 8% of the general female population with a 5% prevalence of high-risk HPVs. In their investigation, they identified cervical cytological abnormalities in 34 women, of whom 35.3% were HPV-positive [115]. The most prevalent subtype identified in the Irani population was HPV16, followed by HPV18. Moreover, while HPV16 was predominant in 60% of invasive cervical cancer, it was present in 9% and 2% of women with abnormal and normal cytology, respectively [115]. Moreover, a pooled prevalence of HPV positivity was reported in ~14% of cervical cancer samples, with a prevalence of 6.5% of high-risk HPV subtypes [73].

As for Israel, studies published from 2017 to 2021 [116,117,118] reported similar results. For instance, Schejter et al., 2021 screened 115,807 cervical samples from the general population and found only 9% positivity for HR-HPV, out of which 37% had abnormal PAP LBC results [118]. Similarly, in patients positive for HIV, HR-HPV was detected in 29% of the screened samples, out of which 35% had CIN 2 or higher on histopathology [117]. In patients with CIN 2-3, HPV was detected in 92.9%, and HR-HPV was detected in 85.8% [116].

Moreover, in Turkey, a pooled HPV prevalence of 8% was reported in cervical cancer samples [73]. The prevalence of high-risk HPV subtypes was 6% [73]. Additionally, Oruc et al. (2022) reported national cervical cancer screening results from 89,302 women between 2015 and 2019. The study found that 94.1% of the samples were HPV-negative, while only 4.9% were HPV-positive. The most common HPV genotypes were 16, 51, 31, and 52 [119].

Overall, studies investigating the prevalence of high-risk HPV-induced cervical cancer in the Middle East and North Africa are still scarce. We believe it is still crucial to conduct larger studies with bigger sample sizes to confirm the incidence of high-risk HPVs in the stated population. Notably, the methodology used for HPV DNA detection varied significantly amongst reviewed papers. Although the majority of studies used RT-PCR [74,79,80,81,82,83,84,92,95,96,99,100,101,102,103], others also used Hybird Capture 2 (HC2) [85], HPV linear array assay and direct sequencing [90], and hybridization reverse blot technique [98,105,106]. The highlighted differences in HPV detection technique used potentially affected the reported prevalence of HPV.

## 3. High-Risk HPVs in Head and Neck Cancer

Head and neck cancers (HNCs) include cancers affecting the oral cavity, pharynx (naso-, oro-, and hypopharynx), larynx, nasal cavity and paranasal sinuses, as well as tumors of salivary glands [120]. HNCs account for 5–50% of all cancers and are the tenth most commonly occurring cancers worldwide [1]. Tobacco consumption is the major risk factor for HNCs, including oral [121]. Viral infections by HPVs are a well-known etiological factor for HNCs, as HPV accounts for approximately 25% of all HNCs [122]. Similar to cervical cancers, HPV16 is the dominant genotype in HNCs; however, genotyping varies based on sex and geographical regions worldwide and ranges from 0 to 60% in prevalence [123]. Jalouli and colleagues [124] analyzed the presence of HPV in oral cavity squamous cell carcinomas (OCSCC) from eight countries in Asia, Africa, Europe, and North America. The study reported that 35% of the samples were positive for HPV; the highest prevalence of HPV was observed in Sudan (65%) [124]. However, due to the limited number of cancer cases from each country (~20 samples), no definite conclusion could be drawn from the study [124]. A recent report examined the prevalence of HPV positivity in oropharyngeal squamous cell carcinoma (OPSCC) in 34 Middle Eastern patients, the majority of them from Lebanon (27), in addition to patients from Syria (3), Iraq (2), Jordan (1), and Palestinian territories (1) [125]. The study reported a comparatively higher HPV prevalence of about 85% in these patients; while the Lebanese patients had HPV positivity of 76%, Syria and Iraq reported HPV positivity of 10% and 7%, respectively [125]. Both Jordan and Palestinian territories reported 3% of HPV positivity [125]. Similarly, a global meta-analysis in OPSCC reported the highest HPV occurrence in Lebanon (85%) [126]. Correspondingly, a recent meta-analysis on HPV-associated HNC prevalence rates in the MENA region reported an overall pooled incidence of 16%; HPV was predominantly present in the salivary glands (20%) and tonsils (16%) [127]. While the study further reported a higher prevalence of HPV-associated HNC in Turkey and Palestine–Israel territory (48% and 31%, respectively), while a low prevalence was reported in Saudi Arabia and Yemen (4% and 2%, respectively) [127]. Additionally, genotyping revealed the predominant presence of HPV16 followed by HPV18 in the MENA region [127]. Below we detail the prevalence and genotype distribution of HR-HPV types and their association with head and neck cancers in MENA countries (Table 2).

### 3.1. In GCC Countries and Yemen

Although there is insufficient literature reporting HPV-associated head and neck cancers from GCC, a few studies were carried out in Saudi Arabia, UAE, and Yemen. However, no similar studies were performed in neighboring countries, such as Bahrain, Kuwait, Oman, and Qatar.

A recent study from Saudi Arabia explored oral tongue squamous cell carcinoma patients, revealing the presence of HPV18 in 23% of the cases. HPV infection was associated with poor overall survival [138]. Another study examined the association of HPV in Saudi patients with head and neck squamous cell carcinoma (HNSCC); the study reported an overall HPV positivity of 3.5% in HNSCC patients, with a predominance in oropharyngeal carcinoma (21%) [139]. On the contrary, Jaber and colleagues [140] failed to report the presence of HPV in oral squamous cell carcinomas.

In UAE, only one study analyzed the presence of the two subtypes, HPV -16 and -18 in the onset and development of oral premalignant and squamous cell carcinoma; the study reported 73% and 71% of the cases to be positive for HPV16 and HPV18, respectively [165]. In addition, 58% of the cases were co-infected with HPV16 and HPV18 [165]. 

Nasher et al. (2014) [160] evaluated the expression of HPVs -16 and -18 in OSCC patients from Yemen; the study did not report the presence of HPVs in the OCSCC samples.

### 3.2. In Levant Countries

In Jordan, to date, only three studies were conducted to analyze the relation between HPV presence and head and neck cancer development. Recently, Khasawneh et al. (2020) [134] reported 31% HPV positivity in head and neck squamous cell carcinoma samples, of which 42%, 37%, and 18% of HPV positivity was present in samples from the oropharynx, oral cavity, and larynx, respectively. On the other hand, while a study on laryngeal squamous cell carcinoma found 15.4% of the samples positive for HPV [135], another investigation focusing on both oral and laryngeal squamous cell carcinomas demonstrated an overall pooled HPV positivity of 15%; compared with laryngeal carcinoma, oral carcinomas exhibited a higher HPV positivity (6% vs. 20%) [152]. Nevertheless, all three studies reported predominance of the HPV16 genotype in their samples (31–85%) [134,135,152].

On the other hand, although the highest HPV prevalence in the MENA region was reported in Lebanon (85%) based on a recent meta-analysis [125,126], only one study in the Lebanese population reported an HPV prevalence of 27% in oropharyngeal squamous cell carcinoma (OPSCC) samples [148].

Likewise, only one recent study in Syrian patients with head and neck cancer reported the presence of high-risk HPVs in 44% of the cases with HPVs -33. 16, -18, -45, -52, -58, -35, -51, and -31 being frequently expressed [136]. 

### 3.3. In Maghreb Countries

Compared with GCC and Levant regions, studies within Maghreb countries are very scarce and limited to two studies from Algeria and Tunisia.

The Algerian study investigated the presence of HPV in different cancers, including HNC. However, the study did not detect the presence of HPV in head and neck cancer [105].

On the other hand, in Tunisia, only one recent study documented the presence of HPV in HNC and reported 56% HPV positivity [133].

The above studies indicate the lack of information and hence warrant the necessity for further investigation.

### 3.4. In Other Countries of the MENA Region and Turkey

Other countries in the MENA region also explore the genotype distribution of HPVs in human cancers, especially Iran and Turkey. There are numerous studies carried out in these countries highlighting a probable association of HPV in the pathogenesis of HNCs. 

An early study in Iran reported 41% HPV prevalence in oral squamous cell carcinoma, with HPV16 as the most predominant, followed by HPV18; however, HPVs -31 and -33 were undetected [166]. Recently, the IROPICAN study, a large multicenter case-control study in Iran, screened 21 α-HPV, 46 β-HPVs, and 52 γ-HPVs using bead-based HPV genotyping assays in 498 head and neck squamous cell carcinoma samples and 242 controls [171]. The study revealed the presence of α-HPVs in only 1.2% of the patients and 2.9% of the controls, from which HPV16 was the most prevalent type among participants [171]. On the other hand, β-HPVs and γ-HPVs were detected in 43.8% and 26.1% of the patients, versus 38.6% and 24.7% of the controls, respectively [171]. Similar to this study, a previous study in 2017 also reported a low HPV prevalence (3%) in head and neck squamous cell carcinomas [149]. In contrast, other investigations revealed a higher HPV positivity (7–43%) in head and neck squamous cell carcinomas [128,129,130,162], with HPVs -18, -52, and -61 as dominant subtypes [130,162]. Additionally, a systematic review and meta-analysis revealed an overall presence of HPV in oral lesions (21%), whereas, in oral squamous cell carcinoma, HPV prevalence was 31% [172]. Moreover, in laryngeal squamous cell carcinomas, 28% of the samples were HPV-positive; HPV16 was predominant, followed by HPV18 [150]. A study by Roshan et al. did not detect the presence of high-risk HPVs in laryngeal cancer [159]. Additionally, in sinonasal inverted papilloma, while benign samples showed 19% HPV positivity with the predominance of low-risk HPVs (HPV 6/11), squamous cell carcinoma cases were all positive for HPV (100%), with high-risk HPVs (HPV16/18) being in high proportion [156].

A Turkish study on 81 patients documented an increase in the prevalence of HPV-associated oropharyngeal cancers from 38 to 70% between the periods of 1996–2003 and 2004–2011 [173], While a very recent study by Köksal et al. (2021) reported a 24.5% HPV positivity in head and neck squamous cell carcinoma cases with a high prevalence of HPV16 genotype (85%) [131]. An earlier study carried out chromogenic in situ hybridization analysis for HPV DNA on fine-needle aspiration materials from metastatic lesions of 26 patients with HNSCC and reported 15% HPV positivity; the study reported HPV-positive tumors in two laryngeal sites, one nasopharyngeal site, and one oral cavity site [169]. In addition, cytologic review of these lesions revealed the presence of both keratinized and non-keratinized metastatic tumors; all HPV DNA-positive HNSCC tumors displayed non-keratinizing characteristics [169]. In general, when it comes to laryngeal carcinomas, reports vary with regards to HPV prevalence from 2 to 41% [142,153,167,168] with HPV16 as the most common subtype [142,153]. In contrast, Celebi et al. did not detect the presence of HPV neither in normal laryngeal mucosa nor laryngeal carcinoma patients [144]. In esophageal carcinoma, HPV DNA positivity was reported in ~10% of the samples, with HPVs -16 and -39 being common [157]. 

It is clear from the above information that more investigations are necessary to confirm the presence and genotype distribution of HPVs in HN cancer cases in the MENA region.

## 4. High-Risk HPVs in Colorectal Cancer

The incidence of colorectal cancer (CRC) increased exponentially in the past years; CRC is the third most common type of cancer and the second leading cause of cancer-related deaths worldwide [1]. Notably, CRC is linked to both hereditary and environmental risk factors, including viral infection by high-risk HPV, EBV, HCV, HBV, HHV8, and HIV [18,174]. According to a report by Donà and colleagues [175], HPV associated CRC is the most common in homosexual men involved in anal sex (MSM), especially amongst HIV-infected individuals receiving treatment. In addition, a number of studies highlighted that HPV infection is the major cause of development of anal/rectal lesions that subsequently develop into CRC [176,177]. Despite the global rise in the number of studies linking HPV to the development and progression of CRC, only a few studies from the MENA region reported data on HPV-associated CRC. These include countries such as Egypt, Iran, Israel, Lebanon, Saudi Arabia, Syria, and Turkey (Table 3). Below we detail the prevalence and genotype distribution of HR-HPV types and their association with CRC in MENA countries.

### 4.1. In GCC Countries and Yemen

Studies demonstrating the association between HPV presence and the onset of CRC in GCC countries are very rare. Only a few studies in Saudi Arabia reported the presence of HPV in CRC. An investigation involving 132 CRC patients in Saudi Arabia reported only two HPV-positive cases (1.5%) [195]. However, another study reported no presence of HPV-16 nor HPV-18 in CRC samples [196]. Data from these studies indicate that HPV prevalence in CRC may be reasonably low within the GCC countries as compared to the rest of the MENA region. 

### 4.2. In Levant Countries

There are comparatively more reports about the presence of HPV in CRC cases in the Levant region.

One very recent study in Iraq identified HPV16/18 positivity in 44.4% of CRC cases using chromogenic in situ hybridization [193].

In Lebanon, our group reported HPV prevalence in 60 cases out of the 94 CRC samples (64%), with HPV-16 and HPV-18 as the most frequent HR-HPV subtypes [17].

We also demonstrated HPV association with CRC development in Syrian cases in two different investigations, with an HPV prevalence of 54% and 37% in each one [53] [192]. Both studies revealed HPVs -16 and -18 as the most frequent HR-HPV types [53].

### 4.3. In Maghreb Countries

Interestingly, to date, there are no studies about the prevalence of HPVs in CRC in Maghreb countries within the MENA region.

### 4.4. In Other Countries of the MENA Region and Turkey

Compared to studies in the GCC, Levant, and Maghreb countries, there are several reports from Egypt, Iran, Israel, and Turkey analyzing the prevalence of HPV in CRC.

There is only one study from Egypt regarding HPV’s association with CRC [197]. The study analyzed the presence of HPV-16/18 in 40 CRC samples, demonstrating the presence of HPV in six cases (15%). The most common HPV subtypes were HPV18 (12.5%) and HPV16 (2.5%); however, none of the 40 CRC cases were co-infected by HPV16/18 [197].

The bulk of the reports on HPV association with the pathogenesis of colorectal cancer in the MENA region come from Iran. Only a few of these studies report a high prevalence of HR-HPVs in colorectal cancer. In this context, a recent study by Tavakolian et al. (2020) reported that 12% of CRC samples were positive for HPV DNA [179]. Another study reported the presence of HPV-16 (10.5%) and HPV-18 (23.65%) in CRC cases [178]. A significantly high prevalence of HPV DNA (83%) was reported in CRC cases with a high frequency of the HPV subtype 16 (68%) [180]. However, the majority of such studies from Iran report a considerably low frequency of HPV association with CRC. An evaluation of 66 CRC patient samples confirmed the association of HPV in only 3 (4%) of these samples [182]. Another investigation reported a frequency of 6% of HPV DNA in CRC; with HPVs -51 and -56 as the most frequent (15.5%), followed by HPVs -16, -18, and -58 (10%), while, HPVs -31 and -33 had the lowest frequency (7.1%) [181]. In accordance with these results, other studies also reported fairly low HR-HPV frequencies. A study by Ranjbar and colleagues [184] reported HPV DNA in 6.25% of CRC cases, of which 7% of the cases had colon cancer while 4% had rectum cancer. Furthermore, HPV-18 was detected as the most frequent type [184]. Likewise, HPV was found in ~3% of 70 adenocarcinoma colorectal tissues and 6% of adenomatous colorectal tissues, with HPV-16 as the most predominant genotype, followed by HPV-18 [183]. In another report, a lower HPV DNA prevalence in CRC patients was presented with only 1 as HPV-positive out of 100 CRC cases [185]. Moreover, Nosrati et al. did not detect any positive HPV cases in their cohort of 50 CRC samples [198]. Although the association of HPV in colorectal cancer may seem to be ambiguous in the Iranian population, an evident trend is observed in the gradually increasing number of HPV-positive cases, this may be due to recent developments in techniques used to detect HPV DNA in general.

On the other hand, data depicting the prevalence of HPV-association with CRC in an Israeli population are part of a large international study on populations from three different countries that included 106 patients from Israel [194]. As stated in this study, all samples were negative for all types of HPV. Therefore, it was concluded that there was no association between HPV and CRC [194].

Conversely, there are numerous studies regarding HPV association with CRC from Turkey. Five studies attempted to evaluate the presence of HPVs in the pathogenesis of CRC. Interestingly, two out of these studies reported the highest frequency of HR-HPV in CRC in the MENA region (81% and 82%) [187,188]; genotyping reported HPVs -18 and -33 as the most frequently detected types of HPVs in CRC tissues [187]. In addition to this, another study reported a considerably high prevalence of HPV-18 (76.4%) and HPV-33 (58.8%) genotypes in the Turkish population [189]. However, other studies from Turkey reported a comparatively low prevalence of HPV association with CRC. While one such study reported the detection of HPV in only 5 (7%) out of 72 colonoscopic biopsies [190], another research group from Turkey reported the complete absence of HPV genotypes among 106 colorectal carcinomas and 62 colon adenomas [191].

Evidently, HPV-16 and HPV-18 are the most commonly associated with CRC among the countries in the MENA region. However, it is of interest to note the high prevalence of certain regional strains of HPV, such as HPV-18 and HPV-33, which were largely identified by three out of five studies from Turkey. Another interesting finding pertains to co-infection cases with more than one HR-HPV type, as was noted by a few groups within the MENA region [53,178,189].

## 5. High-Risk HPVs in Breast Cancer

GLOBOCAN 2020 estimates breast cancer (BC) to be the most commonly diagnosed cancer, accounting for about 2.3 million new cases (11.7%) with a mortality rate of 6.9% globally [1]. As pointed out in our previous paper [199], the association between HPV and BC still generates substantial controversy. For instance, Khodabandehlou, N. et al., 2019, detected HPV DNA in 48.6% of BC samples vs. 16.1% in controls [200]. Such findings were supported by various similar reports [201,202]. Nevertheless, opposing studies reported a lack of HPV DNA in breast cancer samples, proposing the implausible association between HPV and the development of breast carcinomas [203,204]. Notably, some studies suggested that the prevalence of HPV in breast cancer is dependent on geographical locations [205]. As for the Middle East and North African region, studies were limited to Syria, Turkey, and Tunisia. Recent reports also included Algeria, Egypt, Iran, Iraq, Jordan, Lebanon, Morocco, Pakistan, Qatar, Syria, and Turkey (Table 4). Herein, we review all these studies.

### 5.1. In GCC Countries and Yemen

In the State of Qatar, Sher et al., 2020 [216] examined a cohort of 150 freshly obtained breast tissue specimens (50 benign breast lesions, 50 breast cancer, and 50 normal breast tissues) and reported the presence of high-risk HPV in 10% of the subjects with breast cancer, of which 12.12% were invasive carcinomas [216]. Concomitantly, our group examined 74 cases of breast cancer from 2008 to 2019 that were treated surgically in Hamad General Hospital, Qatar, and detected high-risk HPVs in 65% of the cases with a significant correlation between HPV+ breast cancer and triple-negative breast cancer subtype [217].

### 5.2. In Levant Countries

As compared to studies in the GCC, there are comparatively more studies in the Levant region analyzing the prevalence of HPV in breast cancer.

In Iraq, the frequency of HPV in breast cancer was evaluated by detecting the presence of HPV16 E7 protein; they reported an incidence rate of 30.67% in breast cancer patients [212].

Moreover, in Jordan, a recent study by Al Hamad et al., 2020 [213] examined the presence of multiple viruses, including HPV, in Jordanian women with breast cancer; this study revealed that HPV was detected in 21% out of 100 samples of sporadic breast cancers.

On the other hand, in Lebanon, our group conducted a study to determine the presence of high-risk HPVs in breast cancer and normal breast samples [214]. The study reported a comparatively higher prevalence of high-risk HPVs in breast cancer samples (65%), while normal breast samples revealed high-risk HPVs in 36% of the cases [214]. In addition, the study reported HPV52 as the most prevalent high-risk HPV subtype (65%), followed by HPV35, HPV58, HPV45, HPV16, and HPV51 [214]. Interestingly, high-risk HPV types -18, -31, -33, -39, -56, -59, -66, and -68 were not detected [214].

Similar to the Lebanese population, our group explored, for the first time, the genotype distribution of HPVs in BC samples from Syria. In this study, the authors analyzed the expression of high-risk HPVs, as well as correlated E6 oncoprotein and Id-1 gene expression in 113 Syrian breast cancer samples. Using PCR, we were able to establish a high HPV positivity (61%) with HPV33 as the most predominant subtype in this population (56%) [218]. Moreover, 24 of the 113 samples (35%) were coinfected with more than one HPV type [218]. Furthermore, the expression of the E6 oncoprotein correlated with Id-1 overexpression in the majority of invasive breast cancer tissue samples [218]. The study reported an association between infections with high-risk HPVs and human breast cancer progression in Syrian women [218].

### 5.3. In Maghreb Countries

Studies reporting the presence of HPV in breast cancer in Maghreb countries are scarce. In Algeria, HPV DNA was detected in 12% of breast tumor cases, with HPV16 as the most prevalent type accounting for 53.3% of HPV+ tumors [206].

Similarly, in Morocco, ElAmrani et al. examined the prevalence of mucosal and cutaneous HPV in tumors obtained from both controls and breast cancer patients [215]. The study reported HPV positivity in 25% of breast cancer cases and 8% of controls [215]. More specifically, the authors established the presence of both beta and gamma HPV types in breast cancer cases (10% and 7%, respectively) [215]. Notably, the study did not detect the presence of HPVs -16 and -18 in the samples; however, high-risk HPVs -51, -52, -58, -59, and -66 were found in ~5% of breast cancer samples [215].

On the contrary, in Tunisian samples, Hachana et al. did not detect any HPV DNA in breast cancer samples [219].

### 5.4. In Other Countries of the MENA Region and Turkey

Contrary to the scarce data obtained from the GCC, Levant, and Maghreb regions, there are numerous studies in other MENA countries and Turkey analyzing the prevalence of HPV in breast cancer.

In Egypt, a very recent study reported differential expression of HPV in FFPE tissue (17%), fresh breast cancer tissue (50%), as well as white blood cells of breast cancer patients (40%) [220]. Another study by Tawfeik et al., 2020 [207] investigated the presence of HPV and immune T cells in Egyptian women diagnosed with breast cancer and reported that 20% of breast cancer cases were positive for high-risk HPVs, while benign lesions were not HPV-positive. On the other hand, another study reported a higher percentage of HPV-16 in non-inflammatory and inflammatory breast cancer cases (76% and 66%, respectively) [221].

Multiple studies were conducted to measure the prevalence of HPV in breast cancer patients in Iran; older studies reported a prevalence of more than 20% [224,225], while recent studies reported a minimal prevalence of 5.55% and 8.2% [181,210]. Recently, HPV DNA was detected in 12% of Iranian breast cancer samples, with HPV18 being the most prevalent subtype [209]. In addition, a comprehensive meta-analysis by Haghshenas et al. [208], including 11 studies with 1539 Iranian women, reported an estimated HPV prevalence of 23.6%. On the contrary, Jamal et al. [211] reported the complete absence of the HPV genome in 150 samples of breast cancer in Shiraz, Southwest Iran. 

In the Turkish population, the first study by Gumus et al. [222] revealed that 74% of breast tumor tissue is positive for HPV-DNA, along with 32% of normal breast tissue samples. When it comes to their identified genotypes, HPV33 was commonly detected in both tumor and normal tissues (95% and 87%, respectively), followed by HPV18 (54% and 56%, respectively). Furthermore, a recent study by Balci et al., 2019 [223] investigated the presence of HPV in breast cancer and intraductal papilloma; the study revealed a high presence of HPV in both intraductal papilloma and carcinoma (30% and 44%, respectively).

In conclusion, most of the recent studies in the MENA region confirm the presence of HPV in human breast cancer and the effect of geographic locations on it. Therefore, we believe that high-risk HPVs are present and take part in the initiation and/or progression of breast cancers through the interactions of E5, E6, and E7 oncoproteins of HPV viruses, similar to those of cervical and head and neck cancers. However, it is clear that more comprehensive studies with large cohorts of samples are needed to clarify much of the controversy pertaining to HPV association with BC cases. Molecular studies elucidating the specific mechanism of the action of HPV infection in BC carcinogenesis are still nascent.

## 6. Prevention Strategies of HPVs Infection

Since HPV is largely transmitted via sexual activity, appropriate precautionary methods are the mainstay in preventing the spread of the disease. However, a number of prophylactic vaccines were also developed in recent times to prevent infections against both low and high-risk HPV types.

Under normal circumstances, the virus remains generally protected from the functional immune system, specifically antigen-presenting cells (APCs) and patrolling macrophages [226]. However, reports state that the generation of minimal antibody levels is often sufficient in preventing future infections against the same HPV genotype [226]. This is indicative of the fact that HPV prophylactic vaccines that could trigger the generation of neutralizing antibodies against the viral proteins of HPV will indeed offer enhanced prevention. Currently, three HPV prophylactic vaccines are on the market; details about these vaccines and their global use are discussed subsequently (Table 5).

### 6.1. Cervarix^TM^

Cervarix^TM^ is a bivalent HPV vaccine protecting against two HPV types: -16 and -18 [227]. The vaccine contains an equal composition of the L1 proteins of each of these HPV subtypes (16/18). This vaccine is produced in insect cells and formulated in an ASO4 adjuvant comprising alum and a toll-like receptor 4 (TLR4) agonist, MPL (3-O-desacyl-4’-monophosphoryl lipid A) [228]. Cervarix^TM^ is administered through an intra-muscular injection and is usually followed up with repeat doses at 1 and 6 months after the initial dose [226].

Results from the clinical trials of the vaccine indicate that it was highly immunogenic and capable of generating high titers of HPV 16/18 neutralizing antibodies and is well tolerated; thus proving to enhance activity against cervical intraepithelial neoplasia grade 2 (CIN2+) lesions expressing HPVs 16 and 18 [229]. According to another study, the vaccine showed the efficacy of nearly 90.4% against HPV 16 and 18 CIN2+ lesions [230], while providing cross-protection against HPV types 31 and 45, thus increasing its protection against cervical cancer by nearly 80% [231]. Moreover, Cervarix^TM^ maintains an effect over a five years period [231]. On the other hand, as per the results of a study performing trials on participants of varying age groups, higher antibody levels for Cervarix^TM^ were identified in the pre-teen/adolescent group in comparison with the 15 to 25 years age group [232]. This clearly indicates that higher antibody levels found in the younger age group could result in a longer duration of protection against the virus. The currently recommended age for administering Cervarix is 10 to 25 years [228].

### 6.2. Gardasil^®^

Gardasil^®^, a quadrivalent vaccine, is formulated through the generation of virus-like particles (VLPs) for each HPV type through the use of a recombinant *Saccharomyces pombe* vector and offers protection against infection with four HPV types, namely, HPVs -6, -11, -16, and -18 [233]. The purified VLPs for each HPV type, -6, -11, -16, and -18, are combined into 20, 40, 40, and 20 µg per dose, respectively, on an alum adjuvant [226]. Since this vaccine targets both high (HPVs 16 and18) and low (HPVs 6 and 11) risk HPV types, it can be used as a prevention measure against cervical cancers, as well as sexually transmitted diseases (STD), such as genital warts [228]. The vaccine is delivered through an intra-muscular injection as a 0.5 mL dose in a three-shot immunization protocol at 0, 2, and 6 months [226].

### 6.3. Gardasil^®^9

Another FDA-approved HPV-targeted vaccine is Gardasil^®^9. This vaccine is nonavalent and protects against nine HPV types, namely HPVs -6, -11, -18, -31, -33, -45, -52, and -58 [234], making it a much-required evolution over previous HPV vaccines, as it offers protection against a wider range of HPV types, including both high and low-risk HPV groups. Similar to Gardasil^®^, Gardasil^®^9 is formulated on a similar VLP platform and is approved for use in both genders within the age group of 9 to 45 years [234]. The vaccine is able to offer nearly 90% protection against cervical cancer, which is indeed a virtual elimination of the disease in a wider context [235]. In addition, certain studies reported that Gardasil^®^9 also offers a 30% increased prevention of pre-cancer cervical lesions or CIN 2/3, relative to first-generation vaccines [236,237]. Moreover, apart from cancer, this vaccine is also able to provide approximately 75% to 90% prevention against other HPV-associated STDs [237,238].

Although the MENA region is not similar to western civilizations in terms of culture or religion, particularly when it comes to sexual behavior, there are a few social, cultural, and religious factors contributing to vaccine hesitancy [61]. Since the majority of MENA countries are Muslim-dominating ones, identifying the overall conceptions against vaccinations and studying Muslim-specific factors can aid in providing insight into the social and religious factors of vaccine hesitancy in these communities [239]. Various countries in the MENA region experienced a significant increase in vaccine-preventable diseases, including measles and influenza [240]. Concordantly, countries within the MENA region also opposed the initiation of anti-HPV health intervention programs (screening and vaccination) [241]. The majority of these countries saw a spike in the incidence of cervical and other HPV-related cancers, which can be prevented with appropriate vaccination programs. One of the most commonly detected HPV subtypes in the MENA region is HPV16, which is targeted by the currently available vaccines, and hence their use can help to prevent more than 70% of HPV-induced cancer cases in the MENA region. In September 2018, the Ministry of Health and Prevention in the UAE mandated the HPV vaccine for all female students from eighth grade upwards; however, the policy was met with public opposition resulting in the alteration of the Ministry policy to require parent approval prior to vaccination [241].

Evidently, HPV vaccines are well established in terms of their safety and efficacy. However, the applicability of these vaccines is often limited due to a huge gap in awareness [242]. Since HPV is sexually transmitted, such vaccines need to be targeted at adolescents between the ages of 9 and 15 years, typically prior to the initiation of sexual activity. To ensure high vaccine acceptance rates, the MENA region can benefit from campaigns addressing cultural and social concerns, as well as demonstrating safety and vaccine effectiveness.

## 7. Conclusions

In summary, there is sufficient HPV infection burden in MENA countries to warrant public health interventions and to emphasize the need for introducing HPV-based screening strategies and HPV vaccination programs; since it is clear that HPVs are associated with several types of human cancers, including cervical, head and neck, colorectal, and breast. The data presented herein on HPV genotype-specific distribution can provide a reference point to evaluate the efficacy of currently available HPV vaccines and may help healthcare authorities assess the impact of introducing HPV vaccination programs on cancer prevention. Additionally, sexual health education could also protect the MENA population from HPV and other sexually transmitted diseases.

## Figures and Tables

**Table 1:** Prevalence and distribution of HR-HPV and their association with cervical cancer among the MENA countries.

Population (Year)	Country/Region	Cases (n)	Type of Cancer	HPV Prevalence (%)	Reference
2020	MENA region	6104	Cervical cancer	81	[62]
			Abnormal cervical cytology	54	
			General population	16	
2020	Maghreb countries	1001	Cervical cancer	88	[62]
2020	Iran	1138	Cervical cancer	73	[62]
2020	Northeast Africa	302	Abnormal cervical cytology	94	[62]
2020	Northeast Africa	441	Abnormal cervical cytology	31	[62]

**Table 2:** Prevalence of HPV in head and neck cancer in different countries within the MENA region.

Population (Year)	Cases (n)	Cancer	HPV Prevalence (%)	Detection Method	Reference
Iran (2022)	62	HNSCC	12.9%	PCR	[128]
Iran (2021)	108	HNSCC	23.1%	Nested-PCR and overlapping nested-PCR	[129]
Iran (2021)	46	HNSCC	6.5%	PCR	[130]
Turkey (2021)	106	HNSCC	24.5%	CFX96 real-time PCR	[131]
Turkey (2020)	44	Laryngeal carcinoma	2.2%	Multiplex real-time PCR	[132]
Tunisia (2020)	70	Laryngeal carcinoma	55.71%	ISH	[133]
Jordan (2020)	61	HNSCC	31%	Real-time PCR	[134]
Jordan (2020)	52	LSCC	15.4%	PCR	[135]
Syria (2020)	80	HNC	43.7%	PCR and IHC	[136]
Egypt (2020)	92	HNSCC	3.3%	HPV direct flow CHIP system and PCR	[137]
Saudi Arabia (2020)	44	OTSCC	23%	PCR	[138]
Saudi Arabia (2019)	285	OPSCC and HNSCC	3.5% (HNSCC) 21% (OPSCC)	HPV linear-array and RealLine HPV-HCR	[139]
Saudi Arabia (2019)	45	OCSCC	0%	ISH	[140]
Turkey (2019)	53	ESCC	2%	PCR (Aptima Panther system)	[141]
Turkey (2019)	90	Laryngeal carcinoma	12.2%	PCR	[142]
Turkey (2019)	52 (Only 40 samples amplified by PCR)	LSCC	2.5%	PCR	[143]
Turkey (2018)	93 (11 normal laryngeal mucosa and 82 laryngeal cancer)	Laryngeal carcinoma	0% (normal and cancer)	Chromogenic ISH	[144]
Turkey (2018)	82	NPC	1.2%	HPV ISH	[145]
Egypt (2019)	99	OPSCC, lip and tongue SCC (32)	28% (OPSSC) 37% (lip and tongue SCC)	DNA ISH	[146]
Egypt (2018)	126 (70 benign and 56 cases)	LSCC	0% (benign) 3.6% (cases)	PCR	[147]
Lebanon (2018)	30	OPSCC	27%	PCR	[148]
Iran (2017)	156	HNSCC	3.2%	PCR	[149]
Iran (2017)	50	LSCC	28%	PCR	[150]
Egypt (2017)	50	LSCC	18%	IHC	[151]
Jordan (2017)	16	LSCC and OSCC	15% (pooled prevalence6% (LSCC) 20% (OSCC)	Nested PCR	[152]
Turkey (2017)	28	LSCC	26%	Genotyping assay	[153]
Algeria (2016)	10	HNC	0%	InnoLiPA HPV genotyping test	[105]
Iran (2016)	103	ESCC	10.7%	PCR and InnoLiPA	[154]
Iran (2016)	96 (45 controls and 51 cases)	ESCC	44.4% (controls) 31.4% (cases)	Real-time PCR	[155]
Iran (2016)	40 (37 benign and 3 cases)	Sinonasal inverted papilloma	18.9% (benign) 100% (cases)	PCR	[156]
Turkey (2015)	52	Esophageal carcinoma	9.6%	Real-time PCR	[157]
Iran (2014)	30	ESCC	0%	PCR	[158]
Iran (2014)	82 (22 normal and 60 cases)	Laryngeal carcinoma	0%	PCR	[159]
Yemen (2014)	60	OSCC	0%	Taqman quantitative PCR	[160]
Iran (2013)	177	ESCC	27.7%	PCR	[161]
Iran (2012)	14	HNSCC	43%	PCR	[162]
Iran (2012)	177	ESCC	21.6%	PCR	[163]
Iran (2011)	93	ESCC	8.6%	PCR and InnoLiPA	[164]
UAE (2011)	45	OSCC	73.3%	PCR	[165]
Iran (2009)	22	OSCC	41%	PCR	[166]
Turkey (2009)	65	Laryngeal and hypopharyngeal carcinoma	41.5%	PCR	[167]
Turkey (2008)	50	Laryngeal carcinoma	14%	PCR and hybrid capture method	[168]
Turkey (2005)	26	HNSCC	15%	Chromogenic ISH	[169]
Egypt (2005)	50	Esophageal carcinoma	54%	PCR and InnoLiPA	[170]

ESCC: Esophageal squamous cell carcinoma; HNC: head and neck cancer; HNSCC: head and neck squamous cell carcinoma; IHC: immunohistochemistry; ISH: in situ hybridization; LSCC: laryngeal squamous cell carcinoma; NPC: nasopharyngeal carcinoma; OPSCC: oropharyngeal squamous cell carcinoma; OSCC: oral squamous cell carcinoma; OTSCC: oral tongue squamous cell carcinoma; PCR: polymerase chain reaction; and SCC: squamous cell carcinoma.

**Table 3:** Prevalence of HPV in Colorectal cancer in different countries in MENA region.

Country	Cases (n)	HPV Prevalence (%)	Detection Method	Reference
Iran	38	13 (34%)	Nested and semi-quantitative PCR	[178]
	74	9 (12%)	Nested PCR	[179]
	72	60 (83%)	MY/GP nested PCR, P5+/GP6+ auto-nested PCR and direct DNA sequencing	[180]
	84	19 (23%)	q-RTPCR	[181]
	66	3 (4%)	q-RTPCR	[182]
	140	6 (4%)	PCR	[183]
	80	5 (4%)	Nested PCR and sequencing methods	[184]
	100	1 (1%)	PCR	[185]
	50	0 (0%)	PCR	[186]
Turkey	56	46 (82%)	PCR and Southern blot hybridization	[187]
	53	43 (81%)	PCR and Southern blot hybridization	[188]
	67	39 (76%) & 30 (59%)	PCR and Southern blot hybridization/direct DNA sequencing	[189]
	72	5 (7%)	HPV ISH	[190]
	168	0 (0%)	Nested PCR	[191]
Syria	78	42 (54%)	Multiplex PCR and HPV probe array/reverse line-blot assay	[53]
	102	38 (37%)	PCR and IHC	[192]
Iraq	62	(44%)	CISH	[193]
Lebanon	94	60 (64%)	PCR and IHC	[17]
Israel	106	0 (0%)	GP5+/GP6+ PCR reverse line blot method and SPF10 INNO-LiPA method	[194]
Saudi	132	2 (1.5%)	HC2 assay	[195]
	83	0 (0%)	PCR and IHC	[196]
Egypt	40	4 (15%)	Real-time PCR	[197]

CISH: Chromogenic in situ hybridization; HC2: Hybrid Capture 2; IHC: immunohistochemistry; ISH: in situ hybridization; LSCC: laryngeal squamous cell carcinoma; NPC: nasopharyngeal carcinoma; OPSCC: oropharyngeal squamous cell carcinoma; OSCC: oral squamous cell carcinoma; OTSCC: oral tongue squamous cell carcinoma; PCR: polymerase chain reaction; and q-RTPCR: qualitative real time polymerase chain reaction.

**Table 4:** Prevalence of HPV in breast cancer in different countries in MENA region.

Country	Sample Type	Cases (n)	HPVs+ (%)	Reference
Algeria	Paraffin	123	17.9	[206]
Egypt	Frozen	20	20	[207]
Iran	Paraffin/Frozen	1539	23.6 (6.7–40.5)	[208]
	Paraffin	59	11.8	[209]
	Paraffin	72	5.55	[210]
	Paraffin	98	8.2	[181]
	Paraffin	150	0	[211]
Iraq	Fresh	150	30.67	[212]
Jordan	Paraffin	100	21	[213]
Lebanon	Paraffin	102	65	[214]
Morocco	Frozen	76	5	[215]
Qatar	Fresh	33	12.12	[216]
	Paraffin	74	65	[217]
Syria	Paraffin	113	61.06	[218]
Tunisia	Paraffin	123	0	[219]
Egypt	Paraffin	40	17	[220]
	WBC	40	40	
	Fresh	40	50	
Egypt	Paraffin	135	Non-inflammatory: 76	[221]
			Inflammatory: 66	
Turkey	Frozen	50	74	[222]
	Paraffin	45	29.6–44.4	[223]

**Table 5:** Three currently available prophylactic HPV vaccines [69,70].

	Bivalent Vaccine	Quadrivalent Vaccine	Nonavalent Vaccine
Brand Name	Cervarix	Gardasil	Gardasil-9
HPV Subtypes	HPV-16, -18	HPV-6, -11, -16, and -18	HPV-6, -11, -16, -18, -31, -33, -45, -52, and -58
Adjuvant System	AS04 adjuvant system in sodium chloride, sodium dihydrogen phosphate dihydrate	Amorphous aluminum hydroxyphosphate sulfate	Amorphous aluminum hydroxyphosphate sulfate
Expression system	Baculovirus-insect cell	Yeast	Yeast
Scheduled dose	0, 1, and 6 months	0, 2, and 6 months	0, 2, and 6 months
Recommended dose	20/20 μg	20/40/40/20 μg	30/40/60/40/20/20/20/20/20 μg

## Data Availability

Not applicable.
